# Validation of a simple sample preparation method for multielement analysis of bovine serum

**DOI:** 10.1371/journal.pone.0211859

**Published:** 2019-02-05

**Authors:** Diego Luna, Marta Miranda, Antonio Humberto Hamad Minervino, Verónica Piñeiro, Carlos Herrero-Latorre, Marta López-Alonso

**Affiliations:** 1 Department of Animal Pathology, Veterinary Faculty, Universidade de Santiago de Compostela, Lugo Spain; 2 Faculty of Veterinary Medicine, Universidad Central del Ecuador, Quito, Ecuador; 3 Department of Anatomy, Animal Production and Clinical Veterinary Sciences, Veterinary Faculty, Universidade de Santiago de Compostela, Lugo Spain; 4 Laboratory of Animal Health (LARSANA), Federal University of Western Pará (UFOPA), Rua Vera Paz, S/N, Salé, Santarém, PA, Brazil; 5 Elemental Analysis RIAIDT, Lugo, Universidade de Santiago de Compostela, Lugo Spain; 6 Instituto de Investigación e Análises Alimentarias (IIAA), Departamento de Química Analítica, Nutrición e Bromatoloxía, Facultade de Ciencias, Universidade de Santiago de Compostela, Lugo, Spain; Universita degli Studi di Napoli Federico II, ITALY

## Abstract

Here we propose a single acid digestion (SAD) sample preparation method for ICP-MS analysis of animal serum samples to determine trace element contents. The method was evaluated in comparison with a commonly used procedure involving dilution of samples in an alkaline solution (AKD). In the SAD procedure, aliquots (1 mL) of bovine serum samples were treated at low temperature with a mixture of concentrated nitric acid and hydrogen peroxide. Trace elements (As, B, Ba, Cd, Co, Cr, Cu, Fe, Hg, Li, Mn, Mo, Ni, Pb, Sb, Se, Sr, U, and Zn) were directly determined by ICP-MS analysis of diluted solutions of samples. Both methods were sufficiently sensitive to enable quantification of most trace elements, with the exception of the AKD method for Cd, Hg and Pb. The quality of the data was verified by using certified reference material. Good results were obtained for the SAD procedure and all elements, but recoveries were unacceptable with the AKD procedure for Se (recovery: 57%), Cd (154%) and Fe (139%). Strong associations (R^2^>0.90, *P* = 0.000) between the data obtained by both methods were demonstrated for the elements considered. The proposed SAD sample preparation method produced satisfactory results for determining most toxic and essential trace elements targeted in monitoring studies.

## Introduction

Trace element determination in biological and environmental samples continues to be a challenge due to the low concentrations involved and the undesired matrix effects produced by other sample components. In addition to the elements commonly considered toxic (namely As, Cd, Pb and Hg), other metals have been identified as potentially toxic, and at least seventeen essential trace elements can be toxic when consumed in excess [1–3]. In both animals and humans, low and/or high trace element concentrations in tissues are directly related to the pathogenesis of numerous diseases; for example, elevated concentrations of Fe and Cu (capable of inducing oxidative damage) are detected in the brain tissues of Alzheimer’s patients and in the brains of humans and animals affected by other neurological disorders [4–6]. Although toxic elements occur naturally, undesirable exposure to such elements is linked to anthropogenic activities, including industrial processes [7,8]. Monitoring programmes are essential to improve knowledge about the effects of these elements on living organisms and to prevent the associated risks. Domestic animals, particularly cattle [9], have proved to be excellent target species for biomonitoring studies. Trace element concentrations in animal tissues yield valuable information about animal health, the environment (particularly for extensively reared animals) and human nutrition (as relevant constituents of the human diet). As multidisciplinary laboratories must include trace element analysis in their routine services, selection and pre-treatment of appropriate samples are critical steps in biomonitoring studies. Blood is particularly useful in such studies as it can be obtained by non-lethal sampling. Moreover, although trace element concentrations are higher in tissues such as liver and kidney than in blood, modern atomic spectrometry techniques can determine metal concentrations at ng L^-1^ levels, so that blood is now considered a suitable analytical sample in biological and environmental monitoring studies [7,10–12]. Moreover, information regarding toxic elements in blood is important in clinical diagnostics in both humans and animals.

Trace element concentrations in blood can be measured in whole blood or in serum. Although trace elements are present at lower concentrations in serum than in whole blood (usually more than two times lower [12]), most available information on the values of these elements in healthy individuals mainly refers to serum [13,14]. Measurement of trace elements in blood is less accurate and precise than in serum due to the strong influence of the sample matrix, which is greatly affected by numerous physiological and pathological conditions [15]. Moreover, serum samples are easier to prepare than whole blood or tissues, as complex and time-consuming microwave-assisted acid digestion procedures are not required to eliminate the sample matrix. Thus, serum samples can be prepared by simple dilution procedures. The most commonly used of these is the alkali dilution (AKD) method, which enables minimization of the matrix-derived interference for metal determination in modern multielemental ICP-MS instrumentation [7,12]. However, the AKD method does not completely prevent the risk of clogging the nebulizer, torch injector or sampling interface in ICP-MS measurements, which can greatly reduce the accuracy of the determinations [12], particularly during the analysis of large numbers of samples with low levels of trace element concentrations. In order to prevent such problems, acid digestion of samples in closed vessels in microwave-assisted digestion systems is used (despite being expensive and time-consuming) to determine trace and ultra-trace concentrations of metals in a large numbers of samples or micro-samples [7].

Here, we describe a rapid single acid digestion (SAD) sample preparation procedure and evaluated in comparison with the AKD method for preparation of bovine serum samples prior to ICP-MS-based determination of trace elements.

## Material and methods

### Serum samples

The bovine serum samples used in this study were collected as part of an on-going project on mineral nutrition in cattle. Blood samples (10 mL) were taken from the coccygeal vein (with minimal stress) of cows after the morning milking, during routine visits to the farms. All samples were immediately refrigerated and transported to the laboratory. Serum was obtained within 4 h of collection by centrifuging the blood samples at 3000 RCF for 15 min in 9 mL serum tubes (Vacuette, Z Trace Elements Serum Clot Activator; Greiner bio-one, Kremsmünster, Austria). Triplicate subsamples of serum were stored at -20°C until analysis. The sampling procedure has previously been described in detail [9]. Sample collection and other experiments involving the cattle were all carried out in accordance with the Spanish standards for protection of experimentation animals. The Bioethics Committee of the University of Santiago de Compostela (Spain) evaluated the experimental design and verified and gave permission for the sampling procedures carried out.

### Reagents

All solutions were prepared using ultrapure water of resistance 18 MΩ cm^-1^ (produced using a Milli-Q purification system, Millipore Corp., Bedford, MA). Stock standard solutions of the elements (1000 mg L^-1^) were of ultrapure grade: ICP Multi element standard solution IV certiPUR for B, Ba, Cd, Co, Cr, Cu, Fe, Li, Mn, Ni, Pb, Sr and Zn; and ICP standard certiPUR for Hg and Se were both purchased from Merck (Poole, U.K.). As, Mo, Sb and U standards were obtained from Panreac (Barcelona, Spain).

In the SAD preparation method, the samples were digestion in nitric acid (69%, Hiperpur-Panreac, Barcelona, Spain) and hydrogen peroxide (33% w/v, Panreac, Barcelona, Spain). In the AKD preparation method, the samples were digested in a mixture of ammonia (NH_4_OH, 25%, Merck, Darmstadt, Germany), Triton X100 (Panreac, Barcelona, Spain), anhydrous 1-butanol (Panreac, Barcelona, Spain) and EDTA (Panreac, Barcelona, Spain). The certified reference material (NIST SRM-1598a inorganic constituents in animal serum) used to validate ICP-MS measurements was obtained from the National Institute for Standards and Technology, NIST (Gaithersburg, MD, USA).

Polypropylene tubes used for preparation of samples and standards were soaked in 10% Hiperpur HNO_3_ for at least 24 h and rinsed with deionised water and dried before use. The sample tubes were tested and found to be free of trace elements.

### Sample and standard preparation

In the SAD method, the samples were treated as follows: 1 mL of serum obtained as described in section 2.1 was mixed with 1 mL of concentrated HNO_3_ and 0.5 mL H_2_O_2_ in propylene tubes. The mixture was maintained at 60°C for 2 hours to allow digestion of the samples. The digest thus obtained was diluted by adding 2.5 mL of ultrapure water. The sample solutions were then centrifuged at 2000 rpm for 5 min and subsequently analysed by ICP-MS. In the AKD method, 1 mL of serum was diluted 1:5 by addition of an alkali solution consisting of 2% (w/v) 1-butanol, 0.05% (w/v) EDTA, 0.05% (w/v) triton X-100 and 1% (w/v) NH_4_OH. The mixture was then sonicated for 5 min and centrifuged at 2000 rpm for 5 min before ICP-MS analysis.

The working calibration standard solutions were prepared daily by appropriate dilution of the multi-elemental standards described in section 2.2. For the SAD method, the calibration solutions were prepared in 20% (v/v) nitric acid and internal standards (20 μg L^-1^ of Ge and Tb) were added online at a flow rate of 40 μL min^-1^. The calibration solutions for the AKD method were also prepared by dilution of the multi-elemental standards with the same alkali solution as described above for sample preparation.

### ICP-MS determination

ICP-MS-based multi-element determination was performed in an Agilent 7700x ICP-MS system (Agilent Technologies, Tokyo, Japan) equipped with collision/reaction cell interference reduction technology. The continuous sample introduction system consisted of an autosampler, a Scott double-pass spray chamber (Agilent Technologies, Tokyo Japan), a glass concentric MicroMist nebuliser (Glass Expansion, West Melbourne, Australia), a quartz torch and nickel cones (Agilent Technologies, Tokyo Japan). Elemental concentrations were quantified using a MassHunter Work Station Software for ICP-MS (version A.8.01.01 Agilent Technologies, Inc. 2012, Tokyo, Japan). This system was used for analysis of the sample solutions obtained by both the SAD and the AKD preparation procedures as well as for analysis of the reference sample CRM NIST SRM-1598a. All samples were blank corrected and were analysed in triplicate, with Ge and Tb as internal standards. The ICP-MS parameters, the daily instrumental optimisation conditions and the isotopes selected for determination are summarised in Table 1. Calibration curves (in the concentration range of 0.2 and 10,000 μg L^-1^) were constructed daily by analysis of fresh standard solutions immediately before analysis of the serum samples. In all cases, linear responses were obtained with zero intercept, correlation coefficients higher than 0.999, and a relative standard deviation (RSD) lower than 5%.

**Table 1:** ICP-MS operating conditions.

Instrumental settings	RF Power (W)	1550
	Sample Depth	8
	Plasma Gas flow (L min^-1^)	15
	Carrier Gas (L min^-1^)	1.1
	Nebulizer Pump (rps)	0.1
	Spray chamber Temp (°C)	2
	Dwell time (ms)	
	Replicates	3
	Reaction cell: He Gas (mL min^-1^)	3.6
Analytical masses	^7^Li, ^11^B, ^52^Cr, ^55^Mn, ^56^Fe, ^59^Co, ^60^Ni, ^63^Cu, ^66^Zn, ^75^As, ^78^Se, ^88^Sr, ^95^Mo, ^111^Cd, ^121^Sb, ^137^Ba, ^202^Hg, ^208^Pb, ^238^U,	
Internal standards	^159^Tb, ^72^Ge	
Concentration range calibration standards (μg L^-1^)	Co, Cd, Sb, Hg, U	0.2–10
	Li, Cr, Mn, Ni, As, Se, Sr, Mo, Pb	0.2–100
	B, Fe, Cu, Zn, Ba	10–10000

### Quality control

A quality assurance program was applied to sample preparation with both the SAD and AKD methods. Analytical blanks (prepared following exactly the same procedure as for serum samples) were included in all batches, and the corresponding results were used to calculate the limits of detection (LOD, calculated as 3 times the standard deviation of the blanks) and the limits of quantification (LOQ, calculated as 10 times the standard deviation of the blanks). Analytical accuracy was verified by using CRM Animal serum NIST 1598a and spiked samples at the appropriate concentration levels (up to 2–10 times higher than the normal levels in serum). *Intra-assay* and *inter-assay* precision were measured by 10 repeated analyses of the same sample and by analysis of 10 different preparations of the same sample on different days, respectively.

## Results and discussion

### Calibration and sensitivity

Calibration curves for all the elements analysed in this study were linear (R^2^≥ 0.9999) over a wide range of concentrations, for both the SAD and AKD sample preparation procedures. LOD and LOQ values for the ICP-MS determination based on SAD and AKD preparation procedures as well as trace element concentrations in the analytical blanks are shown in Table 2. The LODs and LOQs of the elements present at the highest concentration in serum samples were generally lower (up to one order of magnitude lower for some elements) for samples processed with the AKD method than those processed with the SAD method. Both procedures were sensitive enough to enable determination of most of the trace elements at the concentrations commonly found in bovine serum samples. However, the SAD method was more sensitive for determination of toxic elements such as Cd, Pb, Sb and Sr, present at trace levels in the serum samples. With the AKD method, these elements were below the LOQ in a high proportion of the samples (between 65 to 100% depending on the element).

**Table 2:** Trace element concentrations in the analytical blanks, limits of detection (LOD) and limits of quantification (LOQ) for the acid digestion (SAD) and alkaline dilution (AKD) sample preparation. All values in (μg L^-1^).

	Analytical blanks		LOD		LOQ	
Element	SAD	AKD	SAD	AKD	SAD	AKD
As	0.038±0.008	0.007±0.001	0.024	0.003	0.121	0.017
B	0.027±0.002	0.094±0.002	0.005	0.007	0.026	0.038
Ba	0.007±0.004	0.026±0.001	0.012	0.004	0.058	0.019
Cd	0.004±0.004	0.017±0.011	0.013	0.033	0.063	0.164
Co	0.006±0.005	0.013±0.003	0.016	0.007	0.081	0.033
Cr	0.076±0.038	0.024±0.001	0.115	0.003	0.576	0.015
Cu	0.050±0.058	0.023±0.004	0.174	0.014	0.871	0.073
Fe	0.364±0.082	0.279±0.031	0.246	0.095	1.230	0.479
Hg	0.042±0.017	0.004±0.003	0.050	0.008	0.248	0.040
Li	0.093±0.063	0.171±0.035	0.189	0.104	0.947	0.519
Mn	0.040±0.051	0.014±0.009	0.154	0.026	0.770	0.130
Mo	0.024±0.028	0.009±0.001	0.084	0.003	0.422	0.015
Ni	0.089±0.043	0.013±0.012	0.129	0.037	0.643	0.185
Pb	0.013±0.010	0.020±0.035	0.030	0.106	0.151	0.531
Sb	0.003±0.001	0.010±0.002	0.003	0.005	0.013	0.025
Se	0.116±0.020	0.026±0.024	0.060	0.071	0.299	0.353
Sr	0.014±0.006	0.016±0.013	0.019	0.039	0.093	0.195
U	0.001±0.000	0.001±0.000	0.001	0.001	0.004	0.007
Zn	0.018±0.006	0.043±0.004	0.017	0.012	0.083	0.062

The trace elements were generally present at low concentrations in the analytical blanks (lower than 0.1 μg L^-1^), indicating the high purity of the chemicals used in both SAD and AKD procedures as well as the absence of contamination of the samples during preparation and analysis. In general, the mean concentrations of most of the elements determined in analytical blanks were several orders of magnitude lower than those commonly present in serum samples. The only exceptions to this pattern were some elements present at very low concentrations in serum samples, such as Cd, Hg and U. Nonetheless, the concentrations of these elements in the analytical blanks were at least one order of magnitude lower than in serum, thus guaranteeing accurate determination after blank correction.

### Accuracy and precision

The accuracy of the ICP-MS method after SAD and AKD sample preparation was assessed by using certified reference material (NIST 1598a) and spiked samples (when the certified value is not available or when the analyte was not detected). The SAD preparation procedure showed better results than the AKD method (Table 3). Recovery of the elements from certified samples prepared using the SAD method (in the range 96–118%) was acceptable, according to AOAC Guidelines for method validation [16] and, for most elements, they were much higher than those in the AKD-treated samples. Recovery of elements from the certified samples prepared using the AKD method was not acceptable for Se (57%), Cd (154%) or Fe (139%) and was also suboptimal for Zn (78%). These results confirm the weakness previously described for the AKD method [7]. The unsatisfactory recovery levels are attributed to polyatomic interference (for of Se, and Zn) and interference arising from adsorption on tubing and/or glass surfaces (for other elements). Selenium has important health implications associated with its anti-inflammatory, anti-tumoral and anti-oxidant properties in both animals and humans [2,17]. Serum levels of this element are high enough to enable identification of deficiency/imbalance, and the poor recovery associated with the AKD method constitutes a serious drawback for the analysis of Se. Moreover, data on Se levels is critical in intensive management of livestock, as dietary supplementation of Se and parenteral administration of Se are routinely carried out [13,18] to reduce the incidence of metritis and ovarian cysts during the post-partum period and to enhance fertility by preventing embryonic death during the first month of gestation [19].

**Table 3:** Result of the accuracy study for the acid digestion (SAD) and alkaline dilution (AKD) sample preparation based on analysis of a certified reference material (Animal Serum NIST 1598a) and spiked samples.

	Animal Serum NIST 1598a			Spiked samples	
	Certified value (μg L^-1^)	accuracy (%)		Recovery (%)	
		SAD	AKD	SAD	AKD
As	(0.3)*	ND	87	88.1±9.4	84.2±6.5
B	—			91.5±8.2	95.8±13.1
Ba	—			89.1±8.4	83.9±4.1
Cd	0.05±0.014	109	154		
Co	1.24±0.07	100	87		
Cr	0.33±0.08	ND	ND	93.8±7.4	46.4±12.0
Cu	1580±90	105	98		
Fe	1680±60	114	139		
Hg	0.32±0.19	90	ND	91.0±4.9	84.2±3.2
Li	—			93.1±5.2	105.1±11.0
Mn	1.78±0.33	108	105		
Mo	5.5±1.0	96	82		
Ni	0.94±0.18	107	119		
Pb	—			87.6±9.3	79.4±5.2
Sb	1.00±0.15	108	106		
Se	134.4±5.8	118	57		
Sr	—			96.1±5.7	99.5±1.2
U	—			96.4±11.2	97.4±27.1
Zn	880±24	108	78		

*In brackets only indicative values. ND: Not detected

Recovery from spiked samples of uncertified elements or elements not usually detected (spiked at concentrations up to 10 times higher than the usual values in serum) was adequate for both methods, with the exception of the AKD method for Cr. This negative aspect was also previously described for Cr analysis in human blood by Barany et al. [7], and was attributed to the high carbon content of the samples. This finding explains the better results obtained with SAD than with AKD because the acid digestion reduces the total carbon content of serum samples.

Results of the evaluation of *intra-assay* (assessed from 10 repetitions of the same sample) and *inter-assay* (evaluated by preparing 10 digest solutions of the same sample in different days) accuracy are presented in Table 4. The SAD sample preparation procedure yielded a higher level of reproducibility than the AKD method. The values of relative standard deviation (RSD) for SAD in the *intra-assay* precision were much lower than for AKD, and levels higher than 5% were only obtained for Li and U. In the *inter-assay* evaluation, the accuracy measures were also better for SAD procedure, with undesirable results for Cr, Hg Pb and U in the AKD procedure. In general, the RSD values were slightly higher for elements present at higher concentrations in serum for SAD than for AKD, although they were generally similar. However, for those elements present at very low concentrations (Ba, Cd, Co, Cr, Hg, Li, Ni, Pb and U) the results obtained by the SAD method were much more accurate.

**Table 4:** Results of the *intra* and *inter*-assay precision (expressed as RSD) for the acid digestion (SAD) and alkaline dilution (AKD) sample preparation procedures.

Element	*Intra*-assay (n = 10)		*Inter*-assay(n = 10)	
	SAD	AKD	SAD	AKD
As	4.6	3.0	3.8	4.2
B	3.1	3.7	4.4	4.0
Ba	4.0	7.6	3.0	2.9
Cd	4.0	16	8.9	21
Co	1.9	7.9	2.6	6.2
Cr	4.69	11	5.4	15
Cu	2.6	2.9	2.4	2.6
Fe	2.0	2.4	2.1	2.3
Hg	8.1	13	9.0	24
Li	8.4	8.7	5.5	8.9
Mn	2.4	4.4	5.1	3.8
Mo	1.1	2.3	1.4	2.2
Ni	4.0	8.2	6.2	10
Pb	2.0	11	5.8	47
Sb	1.5	3.5	2.0	2.9
Se	2.3	2.8	2.8	2.9
Sr	2.0	2.4	1.7	2.2
U	3.02	15	7.9	16
Zn	1.94	2.5	2.4	2.4

### Length of sample preparation step and analytical costs

The length of analytical steps is an important parameter to consider in evaluating sample preparation procedures. The AKD method (approx. 20 min per sample) is faster than the SAD method (2h 10 min per sample) because of the longer digestion step in SAD. However, the length of the digestion step does not require direct involvement of the analyst, who can perform other tasks during this time.

Both sample preparation methods use similar and generally available laboratory material. However, in the AKD procedure additional pure chemical products such as butanol, EDTA, Triton X-100 and NH_4_OH are required, thus increasing the cost of the method. Samples obtained using the SAD method are cleaner and contain fewer particles than those obtained by the AKD method. The length of the washing step between ICP-MS analysis of samples prepared using the AKD method must be increased to prevent blockage of the sample introduction system. The analytical time and gas consumption for ICP-MS measurements are therefore higher with the AKD than the SAD method. In addition, the ICP system must also be cleaned more often after processing AKD-treated samples, thus reducing the sample throughput.

### Analysis of the bovine serum samples

Trace element concentrations in the bovine serum samples prepared by both the SAD and AKD methods were measured under the conditions described in section 2.4 and were compared using regression analysis (see Table 5). The results are consistent with those obtained in studies of accuracy and precision. The analysis revealed a close correlation between the two methods (R^2^>0.90, *P* = 0.000) for those elements which presented appropriate figures of merit in terms of accuracy and precision. However, the linear regression analysis revealed statistically significant differences between the methods for some elements, with much lower concentrations determined with the AKD method: As (59%), Co (23%), Li (52%), Mn (41%) and Zn (47%). By contrast, the concentration of Fe in samples prepared by the SAD procedure was 28% lower than in those prepared using the AKD method. Weaker but still significant associations (R^2^≈0.80 *P* = 0.000) were observed for some elements present at very low concentrations in the serum samples (Ni and U) or for those elements for which the analytical recovery was unsatisfactory (Se in the AKD method). Finally, as expected, no association (*P*>0.05) was observed for Cd, Hg and Pb (because most samples were below the LOQ for the AKD method) or Cr (due to interference from carbon in the AKD analysis).

**Table 5:** Comparison of trace element concentrations (μg L^-1^) in bovine serum (n = 20) by using acid digestion (SAD) and alkaline dilution (AKD) sample preparation.

	SAD			AKD				Linear regression analysis		
	Mean±SD	Median	Range	Mean±SD	Median	Range	R^2^	P	Slope	Intercept
As	3.50±0.09	3.54	(2.72–4.24)	2.52±0.11	2.65	(1.06–3.04)	0.921	0.000	1.590	0.488
B	311±9	307	(227–389)	302±9	311	(209–384)	0.902	0.000	0.914	31.8
Ba	13.2±1.2	12.1	(6.7–25.2)	12.3±1.0	11.4	(6.9–24.1)	0.941	0.000	1.023	-0.340
Cd	0.174±0.020	0.154	(0.082–0.397)	0.134±0.021	0.097	(ND-0.412)	0.012	0.709	—	—
Co	0.383±0.015	0.381	(0.245–0.498)	0.311±0.011	0.312	(0.193–0.397)	0.932	0.000	1.235	-0.003
Cr	4.87±0.45	5.13	(2.34–9.29)	0.977±0.179	0.669	(0.83–2.264)	0.004	0.812	—	—
Cu	686±23	694	(530–869)	658±19	665	(514–792)	0.945	0.000	1.077	-22.3
Fe	1983±132	1976	(1137–2996)	2335±203	2321	(1140–3859)	0.977	0.000	0.722	206
Hg	0.422±0.064	0.337	(ND-1.240)	0.032±0.004	0.031	(ND-0.064)	0.107	0.159	—-	—
Li	15.6±0.8	15.8	(10.8–22.7)	10.8±0.6	10.4	(7.7–15.4)	0.95	0.000	1.524	-1.31
Mn	2.12±0.11	1.95	(1.30–3.26)	1.87±0.07	1.81	(1.19–2.65)	0.958	0.000	1.406	-0.500
Mo	19.6±3.8	14.8	(7.1–78.4)	17.9±3.5	13.3	(6.7–75.0)	0.999	0.000	1.041	4.72
Ni	1.89±0.11	1.87	(1.21–3.04)	0.622±0.032	0.617	(0.435–0.974)	0.768	0.000	2.933	0.001
Pb	1.16±0.21	0.97	(0.265–3.05)	0.148±0.019	0.146	(ND-0.239)	0.016	0.810	—	—
Sb	2.04±0.10	1.89	(0.075–3.03)	2.10±0.11	1.89	(1.56–3.17)	0.953	0.000	0.909	0.126
Se	64.8±3.2	62.2	(39.3–102.3)	29.4±1.4	27.9	(16.0–41.1)	0.812	0.000	2.093	3.28
Sr	117±5	117	(79–173)	114±5	111	(80–165)	0.919	0.000	1.024	0.257
U	0.012±0.001	0.012	(0.008–0.018)	0.015±0.001	0.015	(0.010–0.023)	0.788	0.000	0.704	0.001
Zn	859±50	885	(387–1313)	576±33	582	(260–888)	0.974	0.000	1.467	-11.5

SD: Standard deviation; ND: Not detected

## Conclusions

We developed a simple pre-treatment procedure for bovine serum samples prior to ICP-MS mineral determination based on single acid digestion (SAD). The novelty methodology is very simple and easy to replicate, requires small amount of sample, produces clean and particulate-free solutions and enables long-term storage at room temperature. The proposed sample preparation method enables more accurate and precise determination of most of the toxic and essential trace elements in comparison with the commonly used alkali digestion method. In particular, the newly-developed SAD procedure greatly improved the accuracy of determination for Se and yielded a significant increase in the accuracy of determination of elements present at ultra-trace levels.
